# Overview of Dose Assessment Developments and the Health of Riverside Residents Close to the “Mayak” PA Facilities, Russia

**DOI:** 10.3390/ijerph6010174

**Published:** 2009-01-09

**Authors:** William J.F. Standring, Mark Dowdall, Per Strand

**Affiliations:** Norwegian Radiation Protection Authority, PO Box 55, N-1332 Østerås, Norway E-Mails: mark.dowdall@nrpa.no (M. D.); per.strand@nrpa.no (P. S.)

**Keywords:** Mayak, Dose assessment, River Techa populations

## Abstract

The Norwegian Radiation Protection Authority (NRPA) has been involved in studies related to the Mayak PA and the consequences of activities undertaken at the site for a number of years. This paper strives to present an overview of past and present activities at the Mayak PA and subsequent developments in the quantification of health effects on local populations caused by discharges of radioactive waste into the Techa River. Assessments of doses to affected populations have relied on the development of dose reconstruction techniques for both external and internal doses. Contamination levels are typically inhomogeneous and decrease with increasing distance from the discharge point. Citations made in this paper give a comprehensive, though not exhaustive, basis for further reading about this topic.

## 1. Introduction

The Norwegian Radiation Protection Authority (NRPA) has been involved in studies related to the Mayak Production Association (Mayak PA) and the consequences of activities undertaken at the site for a number of years. The NRPA involvement has included the Joint Norwegian Russian Investigation (JNRI), the Joint Norwegian Russian Expert Group (JNREG) and working on projects like “Time-Dependent Optimalization of Strategies for Countermeasure Use to Reduce Population Radiation Dose and Reclaim Abandoned Land” (RECLAIM, EC funded 1994–1998) and the European Union – Russian collaboration “South Ural Contamination” (SUCON, 1996–1999). This paper strives to present an overview of activities at the Mayak Production Association (Mayak PA) and the subsequent quantification of health effects on specific local populations caused by discharges of radioactive waste into the environment. Research into the radiological consequences of activities at the Mayak PA has been a focus area for cooperation between scientists from the Russian Federation and scientists from Europe, the USA and Japan. Citations made in the paper are intended to give a comprehensive, though not exhaustive, basis for further reading about this topic.

**Figure 1:**
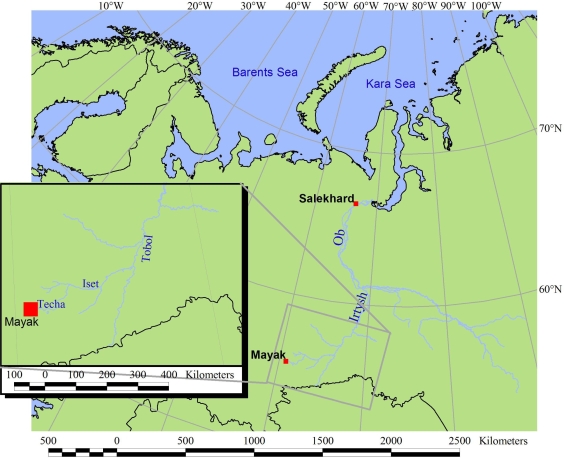
Map showing the Mayak Production Association (inset) and the Techa-Iset-Tobol-Irtysh-Ob river system, draining into the Kara Sea.

Construction of the Mayak PA started in the Chelyabinsk province, close to the town of Ozyorsk (Chelyabinsk-65), in May 1946. The site lies between Yekaterinburg (Sverdlovsk) and Chelyabinsk, east of the Ural Mountains: 55º44′ N 60º54′ E. The Mayak Production Association covers about 200 km^2^ and incorporates the Mayak Chemical Combine, the Chelyabinsk-60 Research Facility and the unfinished South Urals Nuclear Power Plant (NPP). The Mayak PA lies in South Ural, about 1,400 km east of Moscow, close to the River Techa, which forms part of the Techa-Iset-Tobol-Irtysh-Ob river system that eventually drains into the Kara Sea (Figure 1). Today, the Mayak PA facilities include two reactors used for plutonium (^238^Pu) and tritium production; fuel reprocessing facilities; a plutonium processing, finishing, and component manufacturing plant (Plant 20); mixed-oxide (MOX) fuel fabrication plants; fissile material storage and nuclear waste treatment facilities. The Mayak PA had some 15,000 employees in 2000 according to Russian media. Two heavy water reactors (OK-190 and OK-190M) were shut down in 1965 and 1986, respectively. Weapons-grade plutonium production was stopped in 1987. All five of the plant’s uranium-graphite plutonium production reactors (A, IR, AV-1, AV-2 and AV-3) were permanently shut down between 1987 and 1991. Mayak’s activities are currently believed to include reprocessing spent nuclear fuel (SNF), conversion of weapons-grade plutonium into MOX fuel, production of PuO_2_ and UO_2_, production of radioisotopes and the manufacture of electrical devices as well as control and monitoring equipment for pipelines.

### 1.1. The Mayak Facilities

The first uranium graphite reactor (“A-plant”) at the plant became operational in June, 1948; the first batch of plutonium concentrate being produced by the radiochemical facility (“B-plant”) in February, 1949. As of August, 1949, the on-site chemical/metallurgical facility (“V-plant”) had converted the concentrate into weapons’ grade plutonium. The facility then expanded, consisting of seven military nuclear reactors by 1987, at which time weapons’ grade plutonium production ceased. Creation of a nuclear weapon industry raised the problem of radiation safety for the workforce and people living in the vicinity of the plant and general sanitary standards and regulations were adopted in 1948. The Mayak PA was the first production reactor complex built in Russia and has historically been responsible for significant radioactive contamination of the surrounding region.

One important contemporary activity at the Mayak PA is the production of radioisotopes for industrial and medical purposes. Two tritium-producing reactors (Ruslan and Lyudmila: Plant 37) are still in operation; the heavy-water moderated reactor Ruslan was put into operation at the end of 1940s to produce tritium and isotopes for nuclear weapons. It was redesigned towards the end of 80s to a light-water reactor with a capacity of ~1,000 MW. Lyudmila (LF-2) is a ~1,000 MW heavy-water reactor that also produces tritium and various isotopes. Both these reactors have cooling systems utilising water from Lake Kyzyltash (R2). The Mayak PA radioisotope plant was originally known as Plant BB. It now incorporates several additional laboratories and buildings, and is known as Plant 45. It is reportedly a major supplier of radionuclides, radiation sources and radionuclide preparations, having supplied companies in Europe and the USA with a large variety of products including α, β, γ and X-ray sources, fast neutron radiation sources, heat sources (^90^Sr and ^238^Pu based) and a wide range of radioactive isotopes such as ^14^C, ^137^Cs, ^60^Co, ^241^Am, ^238^Pu, ^237^Np, ^193^Ir and ^147^Pm (with specific activities up to ~11 TBq g^–1^ and ~30 TBq g^–1^ for ^60^Co and ^193^Ir, respectively: TBq = 10^12^ Bq).

The first facility at the Mayak PA for reprocessing irradiated fuel, plant (RT-1), was completed in 1977 on the site of the original Facility B. Since then, Mayak has reprocessed spent nuclear fuel (SNF) from VVER-440 PWR, BN-350 and BN-600 fast breeder reactors (FBR), research reactors and nuclear vessel power units. Most reprocessing has been of VVER-440 SNF. RT-1 is equipped with a storage pond for spent fuel, two lines for reprocessing, using a version of the PUREX (purification/extraction) solvent extraction process and one line used for cutting and dissolving fuel. After delivery in specially built railway wagons, SNF is stored in the cooling pond for three years or longer before being chemically processed to separate fuel-grade plutonium and uranium from other waste products, which are destined for vitrification. The facility’s capacity (based on VVER-440 SNF) is projected as 400 t yr^–1^ (t = metric tonne uranium in fuel assemblies). According to Rosatom website data, RT-1 had reprocessed about 3500 t of SNF by 2001.

The Mayak PA also serves as a repository for radioactive materials. Storage can be divided into storage of fissile and/or reactor-grade radioactive materials and the storage of waste products, both “historical” from weapons production and “contemporary” from reprocessing activities. According to inventory estimates made in 1990[1], some 30,000 PBq (PBq = 10^15^ Bq) of solid and liquid waste had been accumulated at the Mayak PA.

### 1.2. Contamination History

Drainage of the area is primarily via the Techa River (Figure 1). In addition, a number of natural lakes and ponds on the plant site have been employed as reservoirs to manage intermediate and low-level radioactive effluents. These include Lake Karachay and Lake Kyzyltash, Reservoirs 3, 4 and 17 (originally local ponds) and artificial Reservoirs 10 and 11 which were created by damming the Techa River. Since the early 1990s, confirmation of nuclear activities at Mayak and information relating to environmental releases of radionuclides has become available in a number of publications [eg., 1–5].

Three significant contamination events have occurred at Mayak PA:

- Direct releases of radionuclides to the Techa (Reservoir 3) between 1949 and 1956.
- The Kyshtym accident – a thermal explosion in a high-level radioactive waste tank in 1957.
- Dispersal of radionuclides from the dried-out bed of Lake Karachay in 1967.

Direct discharges of radionuclides to the Techa river system via sedimentation ponds (Reservoirs 3 and 4) occurred between 1949 and 1956, approximately 98 % of the total activity being released in the period between December 1949 and November 1951. Over 100 PBq of radioactive material was discharged during the whole period [e.g.1, 2, 6], causing severe contamination down the entire length of the Techa River. Ruthenium isotopes (^103^Ru, ^106^Ru) and rare earth nuclides accounted for over 50 % of total activity releases and an estimated 12 PBq ^90^Sr and 13 PBq ^137^Cs were discharged. Alpha releases (including Pu and U isotopes) were lower, amounting to about 2 TBq according to discharge records [1]. Discharges of ^90^Sr and ^137^Cs during the period 1949–1957 contaminated 240 km^2^ of the Techa River floodplain: an area of 80 km^2^ exhibiting concentrations above 3.7×10^10^ Bq km^–2^ [5]. In order to contain the activity and act as a storage basin for low level wastes, construction of dams along the Techa was initiated creating reservoirs containing high levels of radionuclides such as ^137^Cs, ^90^Sr, ^60^Co and plutonium isotopes [1].

A thermal explosion in a tank holding high-level liquid waste (HLW) in September 1957 created what has subsequently become known as the East Urals Radioactive Trace (EURT). Some 740 PBq was released as a result of the accident, an estimated 90 % settling in the immediate vicinity of the site. The remaining activity, about 74 PBq, was released in a plume that is assumed to have reached an altitude of 1 km and which became dispersed by the wind in a NNE direction to form the EURT [1, 7, 8]. The trace, with an initial contamination density of 3,700 Bq m^–2 90^Sr or above (twice that of global fallout), was some 300 km long by 30–50 km wide. The contaminated area was estimated at 15,000–20,000 km^2^, with approximately 100 km^2^ being defined as constituting a serious radiation hazard to humans (>7.4 MBq m^–2 90^Sr: MBq = 10^6^ Bq). The third contamination event occurred between 10 April and 15 May, 1967, when desiccated, contaminated sediments from Lake Karachay were dispersed by wind up to 50–75 km away from the Mayak PA site. An estimated 22 TBq was deposited over 1,800 km^2^, leading to contamination concentrations in the range 11–210 kBq m^–2 137^Cs [1, 9]. Caesium-137 was the predominant long-lived radionuclide dispersed and may have accounted for as much as 75 % of the total radioactive inventory [10].

**Figure 2:**
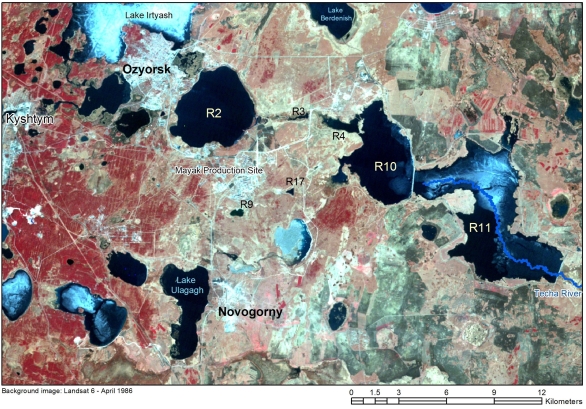
LANDSAT image from 1986 showing the location of different Mayak reservoirs. The blue line through Reservoir 11 traces the original bed of the River Techa before dam 11 was constructed. Lake Karachay is indicated as Reservoir 9 (R9).

The natural and artificial lakes which have been utilized at the site for storing liquid radioactive wastes are shown in Figure 2. Measurements taken in 1993 gave concentrations in water of 70 and 100 MBq l^–1^ for ^90^Sr and ^137^Cs, respectively, in Reservoir 9 (Lake Karachay, R9). Corresponding values for R17 were 300 and 150 kBq l^–1^, respectively [11]. More recent discharges in R9 are documented to be about 50, 27 and 24 PBq in 1994, 1995 and 1996, respectively [11]. Corresponding discharge values into R17 are 21, 4 and 5 PBq of β-emitters. Pollution of groundwater from R9 is a recognised problem. The plume containing R9 contaminates was reported to cover some 10 km^2^ and be spreading at 80–100 m yr^–1^ [1, 11]. Plans exist to fill in and cap R9 with hollow concrete blocks, gravel, and soil and clay layers in an effort to stabilise the site and stop the groundwater contamination plume. Only partial infilling is believed to have occurred to date.

The Techa River Cascade (TRC, Figure 2) is still used for disposal of liquid low-level liquid waste (LLLW). The TRC is comprised of four reservoirs (R3, R4, R10 and R11). The dam for R3 was built in 1951; the dam for R4 already existed but was heightened in 1956; the dam for R10 was built in 1957 and the final R11 dam was constructed in 1964 [1]. Table 1 presents details of the TRC reservoirs.

The water level in the R11 has fluctuated and generally increased in recent years. This is thought to increase the amount of ^90^Sr seepage through dam 11 and into the Techa [12]. Sr-90 concentrations in water samples collected downstream of dam 11 have increased from around 30 Bq/l in 1982 to 90 Bq/l in 2002 [13]. In addition, Asanov Swamp soils and sediments that were contaminated by the early discharges are another main source of radioactivity to Techa waters. The average ^90^Sr concentration in Techa river water sampled at Muslyumovo village (40 km downstream of dam 11) was 6 times the Russian intervention levels in July – August 2004, such that living in this settlement is seen as potentially hazardous to health by the Russian Federal Medical-Biological Agency (FMBA) [13].

### 1.3. Radiation Affected Populations

Population groups exposed to radioactive contamination due to the Mayak PA operations include:

1. The workforce at the Mayak PA
2. Participants in clean-up work carried out after the accidental releases in the Urals
3. Local residents around the Mayak PA who were exposed due to environmental discharges of radioactivity and/or radiation accidents

**Figure 3:**
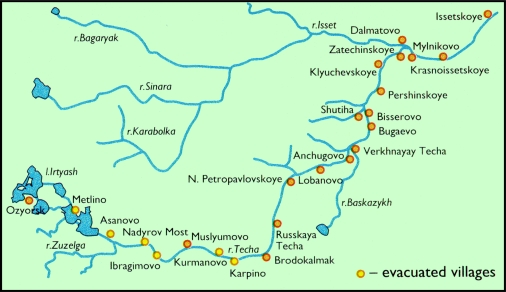
Villages along the Techa River showing those that were evacuated due to radioactive discharges from Mayak PA (Muslyumovo is approximately 50 km from Asanovo) [1].

Members of the third group (offsite population) are the focus of this article and have been subject to detailed studies where personal data on health status in the riverside population groups have been gathered in a registry, the Techa River Cohort. Highest radiation doses were received by residents in riverside villages along the Techa (28,000 people [15]) and those within the initial deposition area of the EURT. The population exposed to Techa River contamination resided in four rural administrative districts (two in the Chelyabinsk and Kurgan Regions, respectively) and consisted of ethnic Russians [Slavic], Tartars and Bashkirs [Asiatic]; Tartars and Bashkirs constituted 24 % of the total exposed population. In 1950, 41 villages (total population: 23,500) were located downstream from Mayak PA when the main discharges occurred. The majority of the settlements were small with less than 500 inhabitants [16]. Four inhabited settlements remain on the Techa River in the Chelyabinsk region, these being (in order of proximity to the Mayak PA): Muslyumovo, Brodokalmak, Russkaya Techa and N. Petropavlovskoye with a total population of 9,229 persons as of the census of 1989 [17]. Of the four remaining inhabited settlements along the river, internal and external dose assessments to the resident population have been studied for Muslyumovo and Brodokalmak in particular. Stretching some 6 km along both banks of the Techa River, the village of Muslyumovo, some 47 km downstream of the industrial reservoirs, had a population of 2,550 in 1995, the majority (97 %) being Bashkir. The village of Brodokalmak stretches 4.5 km along the Techa River, 40 km downstream from Muslyumovo and had a population of 3,700 in 1995, mostly Russian (86 %). Inhabitants of both villages work mainly with local agriculture. About 7,500 people were evacuated from 20 settlements and villages along the Techa River between 1953 and 1960 (Figure 3) due to direct releases, after receiving average effective radiation doses ranging from 35–1,700 mSv [15]. The EURT exposed populations in some districts of the Chelyabinsk, Sverdlovsk and Tyumen Regions (about 20,000 km^2^ with initial depositions over 3.7 kBq m^–2 90^Sr [1]), amounting to 270,000 people in 217 settlements [15]. Approximately 10,200 people were evacuated from the contaminated areas after the Kyshtym accident, the 1,054 residents in the three villages located nearest the accident site receiving the highest doses, with average equivalent doses to red bone marrow of 570 mSv.

The collective effective equivalent dose for the evacuated population amounted to some 1,300 personSv, while it was estimated as being 4,500 personSv for the whole population remaining in EURT territory [15]. The 1967 Karachay incident affected an area covering 63 populated regions, with a total population of 41,500 being encompassed by the 3.7 kBq m^–2 90^Sr isoline. Cs-137 contamination could be divided into two broad areas [1]: 1,800 km^2^ with contamination densities in the range: 11–210 kBq m^–2^ and 34 km^2^ with contamination densities in the range: 210–765 kBq m^–2^. The average individual dose from external radiation from this contamination was estimated as being 13 mSv for 4800 people living closest to Lake Karachay, while doses of 7 mSv were recorded for more distant populations [15].

After the extent of the radioactive contamination of the Techa River became known, countermeasures were implemented to limit doses to the local population, initial prohibitions on water use and evacuation beginning in 1951 along the upper part of the river. Hydrological engineering works such as damming the upper reaches of the river were performed, and wells were dug to supply newly constructed water pipelines. All villages on the upper part of the river, less than 78 km from the site of contamination release (7,500 people), were evacuated between 1953 and 1961 [1]. The effectiveness of the evacuation was however limited, since the riverside village populations had already received their main external and internal radiation dose prior to evacuation. In addition, a sanitary-protection regime was established where the Techa floodlands near villages were fenced-off to prevent the use of river water for drinking and domestic purposes. Fishing, breeding waterfowl and the use of floodlands for cultivation and pasture were abandoned [15]. Restrictions on the use of the Techa and its river water were also introduced along downstream parts of the river in 1956. Some of these restrictions are still in place in the area, including bans on drinking river water, fishing and bathing in the river, access to the river banks and use of riparian pasture for grazing animals. However, no strict control of countermeasures is visibly enforced and several local people are still believed use the Techa River and the riverbank areas to some extent.

After the Kyshtym accident in 1957, several protective measures were put into action in order to reduce population doses: 10,200 people were evacuated from the most contaminated sites at different times after the accident. Some foodstuffs and fodder were discarded and a sanitary control zone was established. In addition, the use of lakes for potable water and fishing was prohibited, pasture and grassland was put under control and the state farms had to change their production profile [15]. Contamination from dried-out radioactive Karachay sediments in 1967 mainly affected areas within the 1957 EURT. Between 1967 and 1971, remediation work was carried out to fill in shallow areas of the lake and to re-cultivate the area surrounding it. The surface area of the lake was reduced from 0.51 km^2^ in 1962 to 0.15 km^2^ in 1994, and this reduction process was planned to continue until the lake was completely filled. At a JNREG meeting in Oslo, November 2005, Russian authorities presented plans to reduce discharges from Mayak PA; according to these plans, discharges to R9 should cease in 2009.

### 1.4. Contamination Levels in Groundwater, Reservoir Water and River Water

Filtered water samples collected from two boreholes in the vicinity of Lake Karachay in 1994 exhibited levels of 4,200 Bq l^–1 60^Co and 8,800 Bq l^–1 90^Sr at depths below 50 m [2], ^137^Cs levels being below 2 Bq l^–1^ at this site. Activity concentrations were considerably lower at a borehole 4 km south of the lake, with maximum levels of 270 Bq l^–1 60^Co and 68 Bq l^–1 90^Sr; data from the regular monitoring of this second borehole showing a steady increase in ^90^Sr levels over the period 1989–1994 [1]. Surface waters sampled from the Mishelyak River, Reservoirs 10 and 11, the by-pass channels and Techa River in 1994 exhibited highest activity concentrations in Reservoir 10 (up to 14 kBq l^–1 90^Sr, approximately 100 Bq l^–1 137^Cs and 2 Bq l^–1 60^Co), with activity levels decreasing in Reservoir 11 (up to 2.4 kBq l^–1 90^Sr, 1.5 Bq l^–1 137^Cs and <0.1 Bq l^–1 60^Co) [1].

Reported concentrations of radionuclides in Techa River water, which primarily receives water from the reservoir cascade via filtration of water through the dam from Reservoir 11 and overland drainage flow from the reservoir by-pass channels, have been relatively constant between the Asanov Swamp and Muslyumovo: levels of 7 to10 Bq l^–1 90^Sr and 0.6 to 0.8 Bq l^–1 137^Cs being reported [1]. Results from similar water samples collected in 1996 were consistent with 1994 data. Data reported in 1998 [18] are also in agreement with these concentrations and show that ^90^Sr concentrations have decreased by a factor of 10^3^ since 1951. Cabianca *et al*., [19] reported low ^137^Cs activity concentrations in Techa River water, (mean 0.15 Bq l^–1^) and mean ^90^Sr activity concentrations of 7.2 Bq l^–1^. Mean activity ^137^Cs and ^90^Sr concentrations were 0.0055 Bq l^–1^ and 0.015 Bq l^–1^, respectively, in drinking water from artesian wells in the Brodokalmak area.

### 1.5. Contamination Levels in Soils

Fieldwork results from JNREG [1] report the highest concentrations of ^90^Sr contamination in soils from EURT sites, ranging from 14 to 34 MBq m^–2 90^Sr. The same group identifies the highest concentrations of ^137^Cs in soils from the Asanov Swamp area, about 7 km downstream from dam 11 where ^137^Cs concentrations were about 42 MBq m^–2^. Contamination patterns in soils are in general heterogeneous [1] and also display differences over smaller areas such as at the towns of Muslyumovo [20], where ^90^Sr and ^137^Cs soil concentrations are reported in the ranges of 600–4810 kBq m^–2^ and 110–1010 kBq m^–2^, respectively, and at Brodokalmak [21].

### 1.6. Contamination Levels in Biota and Food Products

Consumption of local fish has been recognized as an important pathway for human exposures in populations living along the Techa River [22]. Samples of pike filet (Esox lucius) in 1994 from Reservoirs 10 exhibited 80 to 140 kBq kg^–1 137^Cs fw (fresh weight), while whole perch (Perca fluviatilis) contained up to 130 kBq kg^–1 137^Cs. Reported data for pike filet samples from Reservoir 11 indicate levels of 1.3 kBq kg^–1 137^Cs (in 1994), while similar samples collected from the Techa River at Muslyumovo contained 400 Bq kg^–1^ at that time [1]. Kryshev *et al*., [22] reported 450 ± 110 Bq kg^–1^ fw ^90^Sr and 18 ± 8 Bq kg^–1^ fw ^137^Cs for locally caught fish in the riparian villages along the Techa River between 1991 and 1993.

Milk is also very important to exposure levels in local populations, as Techa floodlands are used as pasture for local cows. Kravtsova *et al*., [23] reported from a database containing 1,185 and 1,061 values for ^137^Cs and ^90^Sr contamination in milk, respectively, including data from 1992 to 1998. All milk samples were divided in two groups. The first group included milk produced by cows which had access to the floodplain, the other included cows grazing beyond the floodplain. Mean activity concentrations of ^137^Cs and ^90^Sr were approximately a factor of 10-fold higher in samples from cows grazed on the floodplain, maximum ^137^Cs concentrations being 200 Bq kg^–1^ in Muslyumovo compared to 66 Bq kg^–1^ in Brodokalmak, maximum concentrations for ^90^Sr being 37 and 5.6 Bq kg^–1^. Other studies yield similar results for activities in food products. Cabianca *et al*.,[19] report that activity concentrations of ^90^Sr and ^137^Cs were low (< 10 Bq kg^–1^) in most food products consumed by the population of Brodokalmak, except for fish and in milk from cows grazed on the flood plain. A summary of monitoring data from different studies is provided in Table 2 for ^90^Sr and Table 3 for ^137^Cs.

Data presented in Tables 2 and 3 show that monitoring data exhibit relatively small variations between different studies and temporally. Data for ^90^Sr indicate that activities are quite low in most food stuffs although fish caught in the Techa River are clearly a possible source of ^90^Sr to the local population. Cs-137 is also present at quite low levels in most foodstuffs apart from milk and fish caught in the Techa River. Milk data for cows grazed on non-restricted (i.e. away from the river bank) and restricted pastures show a clear difference for ^137^Cs with averages of 7 Bq kg^–1^ compared to 175 Bq kg^–1^, respectively [19].

## 2. Dose Assessment

The populations living along the Techa River were chronically exposed to radiation, both externally and internally. Villagers were exposed via many different pathways, of which potable water from the Techa River was one of the most significant. External irradiation from the Techa River bottom sediments and shoreline was also an important factor. The river was the main source (sometimes the only source) of water for households in the riverside villages. Techa river water was also given to cattle, used for watering vegetation, breeding waterfowl, fishing, bathing and washing. The radionuclides believed to contribute most to the dose commitment are ^90^Sr and ^137^Cs. A survey of dietary habits of the population of Brodokalmak was carried out in 1996 by the Institute of Plant and Animal Ecology (IPAE), Ekaterinburg [19], investigating the intake of different food products. In 1998, another survey of the dietary habits of the population in Muslyumovo and Brodokalmak was carried out by the Russian Research Institute for Radiation Hygiene (IRH), St. Petersburg [24] in which 71 women and 41 men were polled in Muslyumovo and 14 women and six men in Brodokalmak. The obtained intake rates for adults from both studies correspond quite well with each other and are provided in Table 4, along with earlier estimates from JNRI work [25]. Consumed foods stem mainly from private gardens and local agricultural enterprises in all cases.

In 1967 a systematic program to define fixed cohorts of Techa River residents was initiated [26], about 26,500 people who lived in villages along the Techa River during the period of the highest releases (1949–1952) and for whom residence records were available being enrolled in a cohort now known as the Original Techa River Cohort (OTRC). Over the years, about 5,000 people born prior to 1950 who had moved to one of the riverside villages between 1953 and 1960 were added to the database (“late entrants”). In 1998, these two groups were merged to form the Extended Techa River Cohort (ETRC). In September, 2001, the ETRC included 30,136 people, of whom 4,953 were late entrants [27]. A third cohort consists of about 30,000 children born to exposed parents since 1950: the Techa River Offspring Cohort (TROC). The complete registry of exposed populations living around Mayak PA also contains information on exposed persons in the EURT area. Altogether, the database contains personal data for about 80,000 individuals, 17,000 of whom are from the EURT area. The catchment area for the OTRC included territory of five rural districts in the Chelyabinsk and Kurgan districts through which the Techa River flows, these districts including all of the contaminated villages, as well as all villages where people were evacuated to from the most contaminated villages. Death certificates and other documents about cohort members could be routinely obtained from these areas and the database also contains information such as residence history and results of medical and dosimetric examinations of a population including individuals who have received unusually high doses, but at low to moderate dose rates. It is important to note that attaining the required data for such a large population group is not without difficulty: published results for cohort members will include some cases of incomplete follow-up due to migration, missing records and unknown causes of death. The limitations and future plans for cohort analyses are further discussed in Kossenko *et al*. [27].

### 2.1. Dose Reconstruction

The average effective dose equivalents (representing dose accumulation over 25 years) for persons in the OTRC have been estimated to be from 0.074 Sv to 1.4 Sv, with average absorbed doses to bone marrow estimated as from 0.176 to 1.64 Gy [28]. Initial calculations indicated that individual bone surface doses and external doses could have exceeded 2 Gy [29]. Dose reconstruction methods are undergoing continuing refinement with the development of the Techa River Dosimetry System (TRDS-1996 and 2000) [30]. Reconstruction of the source term and amounts of radioactive material discharged into the Techa River has been very important for dose reconstruction. Operational data like the history of dam construction and watercourse changes have been studied as well as environmental monitoring data to obtain the best possible estimate of the exposure of inhabitants along the river. The reconstructed source term is assumed to be about 100 PBq (10^17^ Bq) of fission products released into the Techa River: most (~98 %) is assumed to have been discharged between the years 1950 and 1951 [1, 31]. However, environmental monitoring data were not available for the first three years of activities at the Mayak PA such that modeling methods have been used to estimate the missing information about discharges. Mokrov [32–33] recently published new assessments about the Techa source term for the early years of discharges of radioactivity, based on hitherto unavailable data from the Mayak PA. The main conclusions were that the accepted discharge inventory of 100 PBq was probably an underestimation of the total amounts of radioactivity discharged from Mayak during this two-year period and that the commonly accepted inventory was biased toward longer-lived radionuclides i.e., a large proportion of the discharges in this period were likely to be radionuclides with relatively short half-lives, as yet unaccounted for in the previous discharge estimates. A scientific workshop to evaluate these findings concluded that while the TRDS-2000 dose reconstruction system was basically sound additional work was necessary such as a re-evaluation of the releases of radioactivity, and that any results based on the TRDS-2000 system should therefore be stated as being preliminary [34].

### 2.2. Internal and External Doses

Sr-90, with it’s half-life of 29.1 years, was the main contributor to internal exposure, as it accumulates in bone tissues and is retained for many years. Therefore, absorbed doses from ^90^Sr in red bone marrow and bone surfaces have been calculated for all age cohorts. As ^90^Sr accumulates in bone tissues, children and adolescents accumulate more ^90^Sr than adults. Reconstruction of internal dose depends on estimates of intake and models for metabolism of ingested radionuclides. In vivo measurements of ^90^Sr in teeth enamel, performed since 1959, have given information about the annual intake of ^90^Sr. Measurements of ^90^Sr whole body content have been necessary for estimating the metabolism of the radionuclide in the human body and thereby find realistic dose conversion factors. Monitoring people living along the Techa River began in 1951, including an extensive program for measuring whole body contents of long-lived radionuclides. Over half of the members of the ETRC have had individual measurements of whole body content of ^90^Sr. In the early 1950’s, analyses involved radiometric measurements of bioassay and autopsy samples. This program continued up to 1993, with over 7,500 analyses performed on bone samples from 5,400 autopsies. *In vivo* measurements started in 1959 by measuring beta activity in front teeth. Since 1974, a special whole body counter has been used to measure whole body contents of ^90^Sr and ^137^Cs [35]. About 30,000 measurements have been collected on more than 14,000 people. Further description of the internal dose reconstruction systems can be found in Degteva *et al*., [35]. Other radionuclides contributing to internal doses were predominantly ^89^Sr and ^137^Cs. Intake rates of ^89^Sr and ^137^Cs have been derived from estimates of ingestion of ^90^Sr scaled in terms of the radionuclide composition of river water. The TRDS database contains age-dependent mean-annual-intake levels for ^89^Sr, ^90^Sr, ^95^Zr, ^95^Nb, ^103^Ru, ^106^Ru, ^137^Cs and ^141,144^Ce [30]. Organ dose conversion factors for ^89^Sr and ^90^Sr were calculated for different time periods after intake using biokinetic models developed from ^90^Sr measurements of Techa River residents. Similar models from ICRP Publication 67 [36] were used for the other radionuclides. TRDS-2000 contains dose-conversion factors for red bone marrow (RBM), bone surface (BS), walls of the upper and lower parts of the large intestine (ULI and LLI), the wall of the small intestine (SI), stomach wall (ST), ovaries, testes and uterus.

Dose rate measurements on the Techa River shoreline have been used to reconstruct external dose to the population along the Techa River [e.g., 25]. Typically, the highest dose rates measured are on the river shoreline and/or on the floodplain areas by the riverside. Absorbed doses due to external exposure can then be estimated on the basis of systematic measurements of gamma dose rates along the banks of the river and the typical living patterns of inhabitants in riverside villages. However, reconstruction of external dose requires information about population behavior, such as time spent indoors and outdoors, what areas are frequented and how often. Here, one should bear in mind that it is difficult to accurately reconstruct behavioral patterns that have occurred 45 to 50 years previously. Several studies have tried to model behavioral factors, where the most important factor was deemed to be time spent on the shoreline. More details can be found in Degteva *et al*., [30]. This approach can give an estimated average annual absorbed dose from external sources for different age groups in each village, but can not provide information on variations in individual doses.

### 2.3. Internal and External Doses Received from 1950 Onwards

Drinking water from the Techa River was the primary pathway for ^90^Sr incorporation in 1950–1951. Later, locally produced milk and fish were the main sources. Internal dose reconstruction has been based on individual measurements of ^90^Sr body contents. ^90^Sr concentrations in the body and tooth enamel were clearly dependent on age. Maximum body contents, and thereby the largest internal doses, were observed in people who were 13–15 years old during 1950–1951; the period with the largest releases. Dietary intake of ^90^Sr in the 1950s and 1960s has been reconstructed from *in vivo* ^90^Sr measurements in teeth and on a model of its accumulation in the enamel [37]. The most important factors determining the intake of ^90^Sr were changes in the radionuclide concentration in the river water, due to dilution, provision of water supply from non-contaminated sources, dietary habits, usage of floodplain lands for pasturing and fodder and the timing and degree of implementation of protective measures. In Muslyumovo, ^90^Sr intake after 1955 was much less than compared to the period 1950–1955. Estimated mean daily intake of ^90^Sr for adult residents were as high as 5,700 Bq day^–1^ in 1950, about 1,200 Bq day^–1^ in 1951 and 1952, decreasing to about 20 Bq day^–1^ in 1956. Estimated daily intakes of ^90^Sr for adults had decreased further to about 2–3 Bq day^–1^ in the 1970s [16].

Correspondingly, the average ^90^Sr content in the skeleton of adult residents from the upper and mid-Techa River region had decreased from about 100 kBq in 1952 to about 8 kBq in 1990. The maximum body burdens of ^90^Sr corresponded with distance from the point of release in the same manner as did the concentration of ^90^Sr in river water, indicating that most of the ^90^Sr was ingested with river water during this period [35]. Highest annual doses were found in bone surface cells, but also the red bone marrow was exposed to significant radiation doses, with over half ETRC members having internal RBM doses of between 0.1 and 0.5 Gy [30]. Since these doses are primarily due to ^90^Sr, earlier calculations have not been significantly altered. Kossenko *et al*., [38] estimated RBM doses of 0–0.049 Gy (21.3 %); 0.05–0.09 Gy (5.2 %); 0.1–0.24 Gy (22.3 %); 0.25–0.49 Gy (17.7 %); 0.5–1.0 Gy (20.3 %) and >1.0 Gy (13.1 %) in the studied population. The highest individual RBM doses were estimated to be 3–4 Gy and riverside residents accumulated 80 % to 95 % of committed lifetime dose within the first ten years of contamination. Internal doses calculated for the GI tract have increased when calculating using the new TRDS-2000 system due the inclusion of short-lived radionuclides [27].

Calculations of external dose for permanent riverside residents during the main releases presented in Degteva *et al*., [30] were significantly lower than earlier assessments published in 1994. The reasons given for this were the adoption of two assumptions during previous calculations: that exposure rates decreased at the same rate downstream as the concentration of beta emitting radionuclides in river water and that external exposures were the same in 1950 and 1951 because approximately the same amounts of radioactivity were assumed to have been released. Analysis of historical monitoring data and modeling radionuclide transport in river water has shown both these assumptions to be wrong, causing overestimates of external doses. The decrease in exposure rate downstream was actually much greater than the decrease in radionuclide concentrations in river water and amounts of radioactivity accumulated in bottom sediments were significantly greater in 1951 compared to 1950 [30]. Further development of external dose reconstruction also included the decrease in doses that occurred with distance from the river banks and a reassessment of behavioral data. The indoor and outdoor dose rates in other areas of the villages were then estimated according to factors like distance from the shoreline and type of area (e.g. building, forest, garden or the street). The new estimates of external dose showed that approximately 80 % of the population received total doses of less than 0.1 Gy, as opposed to “old” calculations where only 25 % received doses less than 0.1 Gy [39].

The distributions of total dose accumulated through 1990 for about 30,000 members of ETRC are presented in Table 5 (RBM - red bone marrow; BS - bone surface; LLI and ULI - walls of the lower and upper parts of the large intestine; SI - the wall of the small intestine; ST - stomach wall).

### 2.4. Current Doses

Early assessments of average intake of ^90^Sr and ^137^Cs in Muslyumovo and Brodokalmak in 1999 are presented in Table 6. Corresponding estimated internal doses for different ages are presented in Table 7 [25].

The internal dose was not estimated for the group using the sanitary zone for food gathering or pasture, but the author gives the assumption that this critical group would have received doses about three times higher than the stated average. Individual internal doses to the population in Brodokalmak are approximately half of the doses received by Muslyumovo residents in this assessment; annual individual internal doses being in the range 10–30 μSv in Muslyumovo and 7–15 μSv in Brodokalmak. The total estimated doses to the population in Muslyumovo and Brodokalmak in 1999, and accumulated up to 1995, are given in Table 8 [25]. For comparison, predicted average annual equivalent external doses in 1999 for children, teenagers and adults in Muslyumovo and Brodokalmak were also calculated. External doses were estimated for normal activity, not using the sanitary zone, and for additional external exposure due to activities within the sanitary zone such as the gathering of wood and hay, linen washing, keeping cattle and waterfowl on the floodplains and especially fishing and recreation by and on the water. In this early assessment, the contribution of the external dose to the total dose is about 90 %.

A more recent study that estimated the current external doses to the population in the Techa area was completed in 1998 [24]. Here, doses received in Muslyumovo and Brodokalmak were also investigated. It was found that residents who frequently use the Techa River and riverside areas receive the highest external doses. Despite official restrictions on economic and domestic use of the riverbank area, the local population still uses the riverside to some extent. Activities performed in the riverbank area included the grazing of cattle and sheep, hunting of waterfowl, fishing, collecting fodder, gathering wood and other materials needed domestically and recreation. To make an initial assessment of external dose, gamma dose rates from man-made radionuclides were measured on the floodplains, in the settlements themselves, and outside the floodplain [24]: mean dose rates were 1520 nGy h^–1^, 15 nGy h^–1^, 22 nGy h^–1^; and 440 nGy h^–1^, 8 nGy h^–1^, 14 nGy h^–1^ for Muslyumovo and Brodokalmak, respectively. Occupancy factor values for floodplains were assessed on the basis of observation data for different age groups during field visits within the settlements. Occupancy factors for other locations (e.g. inside and outside of houses) were based on similar investigations in the Bryansk region with conversion factors of 0.7 Sv Gy^–1^, 0.75 Sv Gy^–1^ and 0.85 Sv Gy^–1^ for adults, school children and pre-school children, respectively.

Total doses calculated in this study are presented in Table 9. Here, the total effective dose is defined as the sum of the committed effective dose due to the ingestion of radionuclides in a year and external effective dose received in that year. Doses assessments were made for three hypothetical groups in the adult population [24]:

- *Group 1.* People, who do not visit the river floodplain, do not consume milk from cows pastured on the floodplain or fish from the river Techa. This population group’s exposure doses are therefore not connected with contamination of the floodplain.
- *Group 2*. People, who visit the river floodplain in accordance with average occupancy factor values (0.03), consume 10% of their average annual milk consumption from cows pastured on the floodplain and 10% of their average annual consumption of fish from the river Techa. This population group receives doses that correspond to the average weighted dose in the settlement.
- *Group 3*. People, who visit the river floodplain with occupancy factor values for herdsmen (0.10), consume 100% of their milk from cows pastured on the river floodplain and 30% of their average annual consumption of fish from the river Techa. This can be defined as the critical population group.

The data in Table 9 shows that the contribution of calculated external exposure to the total dose varied between 40% and 80% depending on the population group, less than compared to earlier assessments [25]. Contributions from ^90^Sr and ^137^Cs to internal doses were similar: the main dietary components contributing to internal dose were fish (29–61%) and milk (11–63%) [24]. Results of this dose assessment suggested that the established dose limit for a population (1 mSv per year) would probably be exceeded by a critical group in Muslyumovo. Further comparison with earlier dose assessments [25] where the lowest dose category (minimum level in Table 8, i.e., are not using riverbank areas for pasture, fishing and recreation) are assumed to compare with Group 1 in Table 9 shows the two dose assessments to compare quite well for low dose populations, though the later assessment [24] indicates a more rapid decline in doses moving downstream from Muslyumovo to Brodokalmak. The later dose assessment [24] predictions for the critical group (Group 3) compared to maximum level predictions made earlier for teenagers who were assumed to use the Techa River flood bank areas most often [25]. The positive effects of restrictions on activities and/or usage of areas closely associated with the Techa River on calculated doses are in agreement with other studies [40].

The most recent published assessment of external and internal doses to Techa residents is based on similar data sets regarding environmental levels of radioactivity [41], consumption patterns and occupancy factors as the studies mentioned here. The main findings were consistent: only a hypothetical critical group in Muslyumovo (equivalent to Group 3, termed as herdsmen) was found to potentially receive more than 1 mSv yr^–1^, the corresponding group in Brodokalmak receiving three times less dose. However, contributions of external doses to the total doses were less than the previous assessments [24–25] being 47 % and 40 % for residents in Muslyumovo and Brodokalmak, respectively [41].

## 3. Health and well-being of riverside residents

The registry of the exposed population serves as the basis for conducting epidemiological cohort studies. The registry of mortality contains information from death certificates retrieved from the State Registrar’s Office archives for the entire population in administrative districts across which the Techa River flows. Altogether, the registry contains information from more than 70,000 death certificates. By cross-matching these certificates with TRC data it has been possible to select more than 6000 death certificates for people who had been exposed along the Techa River and died between 1950 and 1982. These data have made it possible to study possible health effects caused by the contamination in the Techa River [e.g., 27–28]. Epidemiological studies of Techa River residents also offer interesting new insight such as the protracted exposure at low dose rates, a wide range of doses, inclusion of both males and females, ethnicity variation and the long-term follow-up documentation [26].

### 3.1. Potential Health Effects

There is circumstantial evidence to indicate that DNA in chromosomes is the principal target for the biological effects of radiation [e.g., 42–44] and that a connection exists between biological damage and radiation energy absorbed, or dose. Two types of damage can occur when a cell is irradiated. Cell death is usually observed soon after irradiation at high doses. The cell can also survive being irradiated and sometimes will reproduce itself in an altered form due to the radiation energy absorbed. Such a transformation of the cell can result in the formation of cancer cells and genetic damage. The effects of such cell transformation may take years to materialize and are therefore sometimes called “late effects” of radiation.

Several studies of health effects in different populations exposed to ionising radiation have been performed. Population groups studied include survivors after the detonation of atomic bombs over Japan in the Second World War, residents near nuclear plants and nuclear weapons production facilities and individuals exposed to fallout from nuclear weapons testing [e.g., 45]. Studies of survivors after the nuclear bombs detonated in Japan have shown a significant dose dependent increase in the risk of cancers in the stomach, colon, lung, breast, ovary, urinary bladder and thyroid gland. Among persons who were under the age of 20 years at the time of the bomb there was also an increased risk of tumours in neural tissue, excluding the brain [46]. Leukemia was one of the earliest effects seen and remains one of the most striking findings from the follow-up study of the atomic bomb survivors: a significant dependence between radiation dose and leukemia was established. An increased risk for lymphoma was also observed among men. The excess in leukemia mortality has declined with time, while excess deaths still increase with time for cancers other than leukemia [46].

In addition to various types of cancer, chronic radiation sickness (CRS) has also been suggested as having detrimental health effects on local residents around the Mayak PA. Dr. A.K. Guskova and Dr. G.D. Baysogolov first described CRS in several hundred workers at the Mayak PA: it was later diagnosed in riverside residents during the early 1950s [47]. Little agreement on what specific symptoms arise from chronic exposure to doses approaching 0.5 to 1.0 Gy yr^–1^ was found in the wider scientific community, though a key symptom was impairment of the hemopoietic system (formation of blood cells) often accompanied with neurological and immune disorders and hypotension. According to Kossenko *et al*., [45] no new cases of CRS have been diagnosed in recent years and symptoms declined when patients were removed from chronic exposure environments, usually resulting in recovery.

### 3.2. Observed Health Effects in the Techa River Area

Kossenko and Degteva [28] reported an increase in total cancer mortality for riverside residents during the period 1950–1982 compared to unexposed residents in the same region with significant differences between the two ethnic groups, Russians and Tartar-Bashkir, in both exposed and unexposed groups. Buldakov [48] has also reported the existence of CRS and increased frequencies (up to 2 to 5 times as many cases) of leukopenia, neutropenia, thrombopenia, immuno-suppression and still-births in upper Techa residents. A study in the early 1990’s performed by Kossenko *et al*., [49] showed a significant increase of the incidence of leukemia in the exposed population (OTRC) compared to control groups, with dose estimates based on the reconstruction of external (gamma dose rates) and internal dose (^89^Sr, ^90^Sr and ^137^Cs). The excess cases of leukemia in the study group compared to the controls were primarily acute and chronic granulocytic leukemia with a significant correlation of leukemia, morbidity and mortality with dose being observed. Most of the excess leukemia cases were observed 5–20 years after the contamination started. Further analyses [26] suggested that 40 % of the 50 recorded deaths from leukemia were related to radiation exposures and that the excess rate for leukemia was 0.85 excess cases per 10,000 person-year Gy (95 %, CI: 0.2–1.5) [estimated excess cases were computed as the difference between the observed number of cases and an estimate of the number expected in the absence of exposure]. For doses over 0.5 Gy on RBM (22 % of person-years recorded: person-years were computed through to date of death/loss to follow-up or 31 December 1989), 50 % of leukemia cases were related to exposure. The absolute risk value of leukemia genesis calculated by Kossenko *et al*., [50] of 2.94 per 10,000 person-year Gy was lower than atomic bomb survivors, hypothesized as a possible result of the chronic nature of exposures. A statistically significant increase in leukemia incidence had also been observed in patients diagnosed with CRS, though this increase was not reported to cause a decrease in lifespan [47]. Kossenko *et al*., [27] have also reported a statistically significant dose-response relationship for solid cancers. Results suggested that about 3% of the 969 recorded deaths from solid cancers were associated with radiation exposure with an excess relative risk per Sv of 0.65 (95%, CI: 0.3–1.0). Absolute risk values for solid cancers in OTRC members were comparable to atomic bomb survivors. A more recent study [51] where individual dose estimates were calculated using TRDS-2000 for members of the ETRC who had died from either solid cancers (excluding bone cancer) or leukemia concluded that low-dose rate exposures over longer time periods were associated to increased long-term cancer risks. They report excess relative risk per Gy for solid cancers of 0.92 (95 % CI 0.2; 1.7). For leukemia the corresponding values reported are 4.2 (CI 95 % 2.1; 13) and 6.5 (CI 95 % 1.8; 24), including and excluding lymphocytic leukemia, respectively. Furthermore, they associate about 2.5 % of solid cancer deaths and about 63 % of leukemia deaths to exposure to radiation.

During 1994–1996 a research team of the Siberian Medical University conducted an investigation of chromosome aberrations and whole body doses in the radiation exposed population of four settlements in the Techa River Region. The methods used were chromosome analysis, electron spin resonance (ESR) of tooth enamel and whole body measurements of ^137^Cs and ^90^Sr [52]. Approximately 60 individuals from each of the four most exposed remaining settlements in the Techa River Region participated in the survey. Compared to the control group there was a significant increase in frequencies of chromosome aberrations in the peripheral blood of inhabitants of all the four exposed villages. A high frequency of lymphocytes with dicentric and ring chromosomes was observed, corresponding to whole body activity levels of ^90^Sr measured by the whole body counter. The highest whole body ^90^Sr activity levels and frequency and chromosome aberrations were found in Muslyumovo: highest levels of both chromosome aberrations and whole body ^90^Sr activity were found in adults, born between 1949 and 1957 when the largest exposures took place [52]. This indicates that chromosome aberrations can still be found a long time after irradiation that occurred around the time of birth.

The Techa River Cohort data has also been used to study the malignant neoplasms among the progeny of exposed people [53]. The study group comprised children aged 0–4 years during the period from 1950 to 1954, children aged 0–9 years during the period from 1955 to 1959 and persons aged 0–29 years by the end of the year of the follow-up study. The progeny were divided in 6 groups after the estimated doses to parental gonads. The average doses to parental gonads in the six groups were from 0.032 Gy to 1.3 Gy. The study showed that there was no increased number of cancer deaths in the progeny of exposed persons compared to the persons in the control group. However, the small number of deaths by the time of the study was deemed to make the conclusions regarding dose dependencies rather uncertain. Studies of chromosome aberrations in the child population of Muslyumovo have also been reported [44]. Here, a significant difference between Muslyumovo children (n = 15) and an age-matched control group (n = 11) from an uncontaminated Russian village was reported, where 0.56 ± 0.08 chromosome aberrations per 100 cells were recorded in Muslyumovo compared to 0.29 ± 0.07 in the controls [44].

## 4. Conclusions

Although contamination has greatly reduced since the period of maximum discharges of radioactivity from the Mayak PA, external and internal exposure of Techa riverside residents resulting from the initial discharges are still measurable. The reconstruction of the historic discharge inventory from the Mayak PA has proved to be an important part of dose reconstruction work. Average external dose rates in the settlements are lower than within the sanitary zones, with maximum dose rates observed on the floodplain, though many studies have shown that dose rates are heterogeneous. External dose rates are higher in Muslyumovo compared to Brodokalmak. This is to be expected due to the former’s closer proximity to the source of radioactive discharges. Estimates of the contribution to total doses made by external dose have shown a decreasing trend, with the most recent study assessing a more comparable contribution to dose from external and internal sources than thought previously. A slight increase of external exposures witnessed to residents of Muslyumovo and Brodokalmak in recent years is probably due to increased use of the sanitary zone: today, teenagers receive the highest external doses because they use the floodplain more. However, adult residents, especially those born before 1951, have received the highest accumulated effective doses. It is therefore clear that restrictions on usage of the sanitary zones and floodplain would reduce the current doses to riverside residents. Concentrations of ^90^Sr and ^137^Cs in privately produced agricultural food products around the Muslyumovo and Brodokalmak areas have been reported as being quite low for the most important products (milk, potatoes, vegetables, meat), though average intakes of radionuclides are obviously in excess of unaffected populations due to the local contamination. Average effective internal doses are higher in Muslyumovo compared to Brodokalmak with some studies suggesting that frequent use of the floodplain for food and fodder production increase the intake and dose estimates by about a factor three. An increase in the risk of developing solid carcinoma or leukemia has been demonstrated for the affected populations though it is important to note that residents in the area who were not exposed to the highest contamination period (due to age or migration) are believed to experience an approximate two orders of magnitude lower risk for developing radiation induced cancer than those who lived in the area during the period of intensive discharges.

## Figures and Tables

**Table 1:** TRC areas, volumes and estimated inventories in 1991 [14]. Values in parentheses are JNREG estimates [1].

Reservoir	Area (km^2^)	Volume (m^3^ ×10^6^)	Estimated inventory (TBq)	Percentage total activity in sediments (%)
3	0.5–0.8	0.78	666	85
4	1.3	4.1–4.3	222	70
10	18–19	76.64	4070 (1200)	5 (25)
11	44	215.74	1443 (1300)	40 (60)

**Table 2:** Monitoring data (mean and range) of ^90^Sr activity concentrations in foodstuffs (Bq kg^–1^) in Muslyumovo and Brodokalmak from different studies performed between 1990 and 1998.

Product	Cabianca *et al*., [19]	RECLAIM [24]		Romanov [25]	
	Brodokalmak	Brodokalmak	Muslyumovo	Brodokalmak	Muslyumovo
Milk	3.6 (1–5.6)	0.8 (0.04–5.7)^a^	1.7 (0.04–37)^a^	0.82 (0.4–1.2)^c^	0.60 (0.37–0.74)^b^
					0.80 (0.7–1.0)^c^
	Non-restricted pastures: 3.6 (1–4.9) Flood plain pasture: 3.6 (1.6–5.6)				
Potatoes	5.4 (3.2–9.4)	0.63±0.37	0.65±0.34	0.38 (0.21–0.85)^c^	0.30 (0.15–8.1)^b^
					0.55 (0.27–1.5)^c^
Vegetables	5.2–28.5	<4	<5		
Meat	0.6	<1	<4		
Egg	0.6	1.6±1.3	2.0		
Cereals	1.4 (1.26–1.32)				
Fish					
Techa River	340	150	2.7		
Lakes	45 (41–48)				

Dates: a = (1990–1997), b = (1994–1997), c = (1998)

**Table 3:** Monitoring data (mean and range) of ^137^Cs activity concentrations in foodstuffs (Bq kg^–1^) in Muslyumovo and Brodokalmak from different studies performed between 1990 and 1998.

Product	Cabianca *et al*.,[19] (Brodokalmak)		RECLAIM [24]		Romanov [25]	
	mean	range (n)	Brodokalmak	Muslyumovo	Brodokalmak	Muslyumovo
Milk (all)	55	1.8–230 (7)	4.4 (0.04–292) (n=266)^a^	15.3 (0.04–1890) (n=961)^a^	0.71 (0.4–1.1)^c^	1.3 (0.37–6.7)^b^
Non-restricted pastures:	7.1	1.8–12 (5)				2.8^c^
Floodplain pasture:	175	120–230 (2)				
Potatoes	BDL		0.16±0.10	0.36±0.20	0.74 (0.5–1.4)^c^	0.63 (0.37–6.3)^b^
						1.0 (0.8–1.3)^c^
Vegetables	BDL		<0.4	<4.6		
Meat (poultry and beef)	0.6			<14		
Egg	2.8					
Cereals	1.3	1.26–1.32				
Fish						
Techa River	580			220±48		
Lakes	58	38–92 (3)				

BDL – below detection level; Dates: a = (1990–1997), b = (1994–1997), c = (1998)

**Table 4:** Assumed intake of different food products in Muslyumovo and Brodokalmak (kg y^–1^).

Food product	Cabianca *et al*., [19]		RECLAIM [24]		Romanov [25]	
	Average	Most exposed group	Average	Range	Range (age 1 to > 17 yrs)
Milk	100	423	255	0 – 1280	164 – 197
Meat	25	105	55	0 – 175	9 – 547
Potatoes	91	250	146	26 – 365	46 – 120
Vegetables	80	350	55	0 – 110	15 – 40
Fish	24	100	37	0 – 183	
Bread	130	270			77 – 237
Water	820	1200			24 – 547

**Table 5:** Percentage of about 30,000 ETRC members within different total dose ranges to different organs from 1950 to 1990. (from [39]).

Organ	≤ 1 mGy	1–10 mGy	10–100 mGy	100 mGy–1 Gy	> 1 Gy
RBM	7.9	12	23	55	1.7
BS	9.0	9.5	13	57	11
LLI	11	12	44	34	–
ULI	12	16	54	18	–
SI	14	56	22	7.7	–
ST	14	58	20	7.6	–
Testes	13	59	20	8.2	–
Ovaries	16	58	20	7.1	–
Uterus	15	58	20	7.1	–

**Table 6:** Average annual intakes of ^90^Sr and ^137^Cs for populations in Muslyumovo and Brodokalmak in 1999 (Bq y^–1^). Data are based on privately produced food (adapted from [25]).

Age (y)	Muslyumovo		Brodokalmak	
	^90^Sr	^137^Cs	^90^Sr	^137^Cs
<1	58	310	52	130
1	110	620	100	270
5	170	620	160	270
10	230	680	210	300
15	230	830	210	360
>17	230	930	210	400

**Table 7.** Average individual internal dose to the population in Muslyumovo and Brodokalmak in 1999, and accumulated up to 1999, depending on age (mSv) (adapted from [25]).

Age (1999)	Muslyumovo						Brodokalmak					
	Accumulated up to 1999			Intake in 1999			Accumulated up to 1999			Intake in 1999		
	^90^Sr	^137^Cs	Total	^90^Sr	^137^Cs	Total	^90^Sr	^137^Cs	Total	^90^Sr	^137^Cs	Total
<1				0.00022	0.0065	0.0067				–	–	–
1	0.00042	0.013	0.013	0.00048	0.015	0.016	0.0004	0.0057	0.0061	0.0002	0.0027	0.0029
5	0.0034	0.068	0.071	0.00056	0.012	0.013	0.0032	0.030	0.033	0.0004	0.0065	0.0070
10	0.0087	0.086	0.095	0.00060	0.015	0.016	0.0080	0.038	0.046	0.0005	0.0051	0.0056
15	0.012	0.17	0.18	0.00030	0.029	0.029	0.011	0.074	0.085	0.0004	0.013	0.013
17–18	0.014	0.24	0.25	0.00032	0.036	0.036	0.012	0.10	0.11	0.0002	0.016	0.016
18–48	0.042	1.0	1.1	0.00032	0.036	0.036	0.039	0.44	0.48	0.0002	0.016	0.016

**Table 8:** Total individual external and internal dose to the population in Muslyumovo and Brodokalmak in 1999 and accumulated up to 1995 (mSv). Minimum Levels indicate no use of the sanitary zone; Maximum levels indicate unlimited use of the sanitary zone. Numbers in brackets indicate the contribution of the external dose to the total dose in percent (adapted from [25]).

	Year	Muslyumovo			Brodokalmak		
		Children (> 2y)	Teenagers (12–17)	Adults (18–48)	Children (> 2y)	Teenagers (12–17)	Adults (18–48)
Minimum level	Up to 1995:	0.51 (86)^a^ 1.3 (93)^b^	2.3 (92)	11 (90)	0.20 (84)^a^ 0.52 (91)^b^	0.92 (91)	7.0 (93)
	1999:	0.15 (91)	0.17 (82)	0.15 (75)	0.0061 (90)	0.068 (81)	0.13 (87)
Maximum level	Up to 1995:	3.0 (93)^a^ 7.8 (96)^b^	14.5 (96)	65 (95)	1.2 (92)^a^ 3.1 (96)^b^	5.9 (96)	28 (95)
	1999:	0.78 (94)	1.1 (92)	0.67 (84)	0.32 (94)	0.42 (91)	0.27 (82)

^a^ children 2–7 years;

^b^ children 7–12 years

**Table 9:** Distribution of individual annual doses received by the population of Muslyumovo and Brodokalmak in 1998 (adapted from [24]).

Village	Annual effective dose (mSv)											
	External				Internal				Total			
	mean	geom. Mean	5% conf.	95% conf.	mean	geom. mean	5% conf.	95% conf.	mean	geom. Mean	5% conf.	95% conf.
**Muslyumovo**												
Group1	0.05	0.04	0.03	0.07	0.07	0.06	0.02	0.16	0.12	0.11	0.05	0.21
Group2	0.28	0.23	0.08	0.70	0.09	0.07	0.03	0.19	0.39	0.34	0.15	0.78
Group3	0.89	0.67	0.19	2.34	0.25	0.20	0.06	0.62	1.13	0.93	0.34	2.57
**Brodokalmak**												
Group1	0.03	0.03	0.02	0.04	0.05	0.04	0.01	0.12	0.08	0.07	0.03	0.15
Group2	0.09	0.08	0.03	0.21	0.05	0.04	0.02	0.12	0.15	0.13	0.06	0.29
Group3	0.27	0.21	0.06	0.67	0.10	0.08	0.03	0.20	0.37	0.31	0.12	0.81
